# Impact of nanoscale silicon dioxide coating of stainless-steel surfaces on *Listeria monocytogenes*

**DOI:** 10.1007/s12223-023-01089-1

**Published:** 2023-09-09

**Authors:** Nadja Hillig, Felicitas Schumann-Muck, Ahmad Hamedy, Peggy G. Braun, Martin Koethe

**Affiliations:** Institute of Food Hygiene, Faculty of Veterinary Medicine , Leipzig University, An den Tierkliniken 1, 04103 Leipzig, Germany

**Keywords:** *Listeria monocytogenes*, Cross-contamination, Nanoscale, Coating, Stainless steel

## Abstract

High resistance to environmental factors as well as the ability to form biofilms allow *Listeria monocytogenes* to persist for a long time in difficult-to-reach places in food-producing plants. *L. monocytogenes* enters final products from contaminated surfaces in different areas of plants and poses a health risk to consumer. Modified surfaces are already used in the food industry to prevent cross-contamination. In this study, stainless-steel surfaces were coated with nanoscale silicon dioxide and the effects on attachment, bacterial growth and detachment of *L. monocytogenes* were evaluated. Attachment was considered for three different ways of application to simulate different scenarios of contamination. Bacterial growth of *L. monocytogenes* on the surface was recorded over a period of up to 8 h. Detachment was tested after cleaning inoculated stainless-steel surfaces with heated distilled water or detergent. Coating stainless-steel surfaces with nanoscale silica tends to reduce adherence and increased detachment and does not influence the bacterial growth of *L. monocytogenes*. Further modifications of the coating are necessary for a targeted use in the reduction of *L. monocytogenes* in food-processing plants.

## Introduction

*Listeria monocytogenes* is a psychrotrophic and environmental resistant pathogen posing a public health threat (European Food Safety Authority 2022b). High resistance to environmental factors such as temperature (− 2 to 44 °C), food salinity (up to 10%) and pH (4.5 to 9.5) allows *Listeria* spp. to accumulate in food and be transferred to processing environments for different food types like ready-to-eat salads, raw milk products, smoked fish or meat (European Food Safety Authority 2022a; McClure et al. 1991; Petran and Zottola 2006; Shahamat et al. 1980). This food-associated infection is especially a risk for young, old, pregnant and immunocompromised people (YOPI). With a lethality rate of 9%, listeriosis is one of the most serious notifiable foodborne diseases (European Food Safety Authority 2022b). *L. monocytogenes* is therefore also legally classified as a food safety criterion under Regulation (EC) No 2073/2005. Consequently, there is a low to zero tolerance for the bacterium in all ready-to-eat products depending on the stage where the criterion applies (Anon 2005; Nielsen et al. 2019). A critical issue in reducing the transmission of pathogens through food is the prevention of cross-contamination through processing surfaces and equipment. In order to detect and break through this persistence of *L. monocytogenes* in food plants, it is necessary to take additional preventive measures on top of optimal monitoring with suitable detection methods for given surface conditions. There have already been tests of promising physico-chemical methods such as treating processed meat with atmospheric pressure plasma, pulsed light or UV-C. However, these methods are currently unfeasible in practice (Albert et al. 2021; Lee et al. 2011). Other measures such as the use of bacteriophages for decontaminating food preparation surfaces are currently not authorised in the European Union (German Federal Institute for Risk Assessment 2019; Żbikowska et al. 2020). An easily applicable method would be a coating of food contact materials like stainless steel. The morphology of the coating on the surface structure has a direct impact on the ability of adhesion of bacteria. Depending on the size of the bacterium, the relative size of indentations on the surface should be smaller to reduce adhesion (Whitehead et al. 2005). It has been shown that a nanoscale surface is more difficult to colonise with bacteria than surfaces with coarser surface structures (Friedlander et al. 2013). Various materials have been used for coatings on surfaces: pores of a size of 15 to 25 nm in an aluminum coating made it difficult to attach to the surface and form biofilms (Zakarienė et al. 2018). Other research groups reviewed nanostructured materials like titanium (Ivanova et al. 2010; Puckett et al. 2010; Truong et al. 2009), aluminum (Feng et al. 2014), polyethylene terephthalate (Campoccia et al. 2006), gold (Díaz et al. 2007), silver (Zakarienė et al. 2018) and glass (Mitik-Dineva et al. 2008). These surfaces are used in industries such as orthopaedics to prevent the formation of biofilms in implants or as packaging materials for food (Zakarienė et al. 2018). Silicon dioxide coating is an innovative approach to be tested for this application. Various research groups have already reported reduced attachment of *Listeria* ssp. to nanostructured silica-coated aluminum surfaces (Feng et al. 2014; Hsu et al. 2013) as well as of *S.* Enteritidis and *E. coli* to silica-coated stainless-steel surfaces (Schumann‐Muck et al. 2023).

The aim of the study is therefore to investigate the potential impact of nanoscale silicon coating of stainless-steel surfaces on attachment, growth and detachment of *Listeria monocytogenes* to generally contribute to a reduction of the load of this bacterium in food-processing environments.

## Material and methods

### Bacterial material

*L. monocytogenes* isolate DSM 27575 (serotype 4b) from the master collection of the Institute of Food Hygiene of the Faculty of Veterinary Medicine Leipzig, Germany was used as an experimental strain. It was cryopreserved at − 80 °C (Cryobank, Mast Group Ltd., Germany). Fresh working cultures were established on trypticase soy yeast agar (TSY, Merck KGaA, Germany) and used for experiments for 4 weeks at most. An overnight culture was prepared (in the following named bacterial suspension) by adding a single colony of *L. monocytogenes* from TSY plate to 4.95 mL of tryptone soy broth (sifin diagnostics gmbh, Germany) by means of an inoculation loop, for each experiment. The broth was incubated at 37 °C for 16 h until stationary phase to reach a cell density of approx. 1.0 × 10^9^ cfu/mL. Initial concentration of the bacterial suspensions was determined for each individual experiment. Bovine serum albumin (VWR International GmbH, Germany) was added to a final concentration of 0.3% to achieve a protein contamination (according to DIN EN 13697:2019).

### Surface preparation

Stainless-steel surfaces (GK Formblech GmbH, Germany) are conformed to the specifications of stainless-steel surfaces in slaughterhouses and food-processing plants. Discs of stainless-steel type 304, polished to grade 2B (finish) on both sides, diameter: 20 mm, height: 1 mm and chrome-nickel steel (X5CrNi1810) were used. A surface pre-treatment was carried out according to DIN EN 13697:2019–10 and included degreasing, sterilisation and autoclaving of the stainless-steel discs to produce a sterile test surface. They were decontaminated for 60 min in a 5% Decon90 solution (Decon™, Fisher Scientific GmbH, Germany), washed with distilled water, sterilised for 15 min in a 95% propanol solution (Carl Roth GmbH, Germany), washed again with distilled water and air-dried in a biosafety cabinet. Dried discs were autoclaved at 121 °C and either stored at room temperature or coated with silicon dioxide. Coating was realised by Nanopool GmbH, Germany, who applied their commercially available product Liquid Glass Metal to cover the discs with a nanoscale layer of silicon dioxide.

### Study design

Bacterial attachment, growth and detachment of stainless-steel surfaces were analysed with regard to the coating. Three different types of attachment were carried out four to five times with six uncoated and six coated stainless-steel discs each at room temperature (21 ± 2 °C). First, inoculation by liquid was simulated by pipetting 50 µL of bacterial suspension on six coated and six uncoated discs centrally (approx. 5.0 × 10^7^ cfu/disc). After 5 min of incubation, excess liquid was removed by gently shaking it off two times.

The second attachment type was pressing against surface material by transferring the bacterial suspension through a silicone plug with a height of 30 mm and a diameter of 18 mm. The plug was dipped into 50 µL of undiluted bacterial suspension and subsequently pressed onto a disc for 3 s with a force of 5 kg, monitored with a laboratory balance. An average of 7.9 µL was transferred to the disc (approx. 1.0 × 106 cfu/disc), from which the actual amount of bacterial contamination was calculated. Inoculated discs were air-dried in a biosafety cabinet for 20 min until only a moist film was visible on the surface.

As a third attachment type, the contact by sliding along the equipment surfaces was simulated by using a silicone plug with a height of 20 mm and a diameter of 12.5 mm to apply the bacterial suspension on the discs. A volume of 20 µL of undiluted bacterial suspension (approx. 1 × 107 cfu/disc) was pipetted onto plug. Discs were swiped three times over the inoculated plug surface by using sterile forceps and dried in a biosafety cabinet for 20 min until only a moist film was visible on the surface.

Growth of *L. monocytogenes* was analysed in six experiments over 8 h at 10 and 30 °C. For hourly measurement, three uncoated and three coated stainless-steel discs each were inoculated with 50 µL of diluted bacterial suspension (approx. 5.0 × 10^4^ cfu/disc) and placed in a separate Petri dish. Both uncovered Petri dishes were placed in a polypropylene box (Lock n’ Lock, 1.20 l, EMSA GmbH, Germany) with a small Petri dish containing 50 µL distilled water (for incubation temperature of 30 °C only). The lid of the box was only placed on top but not locked. A humidity logger (EBI 20-T, ebro, Xylem Analytics Germany Sales GmbH & Co. KG, Germany) was placed in the box for the 8-h measurement to control a constant humidity and temperature hourly. The total initial bacterial count was calculated as a baseline to be able to determine an accurate growth or loss rate. Three uncoated and three coated stainless-steel discs were inoculated with 50 µL germ suspension and immediately recultivated without incubation.

Detachment of *L. monocytogenes* was carried out three times with six uncoated and six coated discs each. Analogously to the procedure for attachment experiments, 50 µL of undiluted bacterial suspension (approx. 1.0 × 107 cfu/disc) was pipetted directly onto a horizontal disc, followed by incubation for 5 min and shaking off excessive liquid. The discs were magnetically fixed (disc magnet, diameter: 20 mm, height: 15 mm, neodymium, N42, nickel-plated, Supermagnete GmbH, Germany) to a self-designed triangular framework (length: 6 cm, width: 11 cm, height: 8.5 cm, polyamide (PA12), 3D-printed by 3Faktur GmbH, Germany) to achieve a positioning angle of approximately 50°. The stainless-steel discs were rinsed with 1000 µL of distilled water heated to 80 ± 5 °C and excess liquid droplets were removed by shaking the disc twice. Analogously, inoculated discs were covered with foam of sodium hydroxide-based protein and fat solving detergent (Eiweiß-Fettlöser flüssig, Ernst GmbH & Co. KG, Germany). Foam was prepared by using a 20-mL syringe with attached 5-µm filter (Minisart Syringe Filter, Sartorius Stedium Biotech GmbH, Germany) as follows: 20 mL of a 0.5% detergent solution was aspirated. Half of the volume was dropped under pressure and replaced by air, which was then aspirated. The remaining 10 mL of detergent solution was pushed out except for a small residue covering only the bottom of the syringe. A complete aspiration of the syringe with air under pressure resulted in the formation of foam in the syringe. Application produced sufficient stable foam to cover the entire surface of two discs and was repeated until foam for all discs was generated. Discs were exposed to the foam for 5 min and subsequently immersed twice in hot distilled water (80 ± 5 °C) and excess liquid was shaken off.

### Recovery and enumeration of *L. monocytogenes*

Bacteria were recovered from discs according to DIN EN 13697:2019–10. Discs were placed with the inoculated side down into a 25-mL glass beaker filled with 2.1 g of 3-mm sterile glass beads (VWR International GmbH, Germany) and 10 mL sodium chloride peptone solution (Carl Roth GmbH + Co. KG, Germany). Bacterial cells were removed from disc surface by rotating at 150 rpm for 5 min (Shaker RS-OS 5, Phoenix Instrument GmbH, Germany). Decimal dilutions were spread on TSY agar plates, incubated at 37 °C for 24 h and colonies were counted manually.

### Data analysis

All statistical analyses were performed by Prism9 Software (GraphPad Software, LL, USA). For this, all bacterial counts were log_10_ transformed and actual bacterial concentrations were considered because of slight differences on different experimental days. Differences between uncoated and coated discs per treatment (attachment/detachment) or per time point (growth) were considered statistically significant when the *p* value was < 0.05. The Shapiro–Wilk test was used to check for normal distribution in advance of execution of *t*-tests for mean value comparisons.

For bacterial attachment, the mean values and standard deviations (SD) of four to five repetitions were calculated from the mean differences of the applied and recovered bacterial counts of six discs per repetition. Results were expressed as reduced attachment because the value represents the number of *L. monocytogenes* that was unable to attach to the surface. Growth capacity was calculated as the average of six repetitions from the differences of three discs at a certain time point minus initial bacterial count. Detachment was calculated as the average of three repetitions from the mean differences of the applied and recovered bacterial counts of six discs per repetition after each cleaning method. Detachment values represent the number of bacteria that did not detach.

## Results

### Attachment

**Fig. 1:**
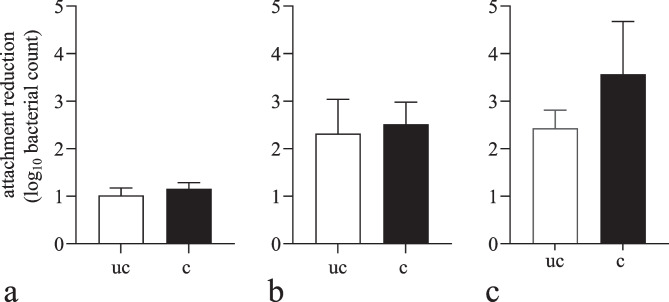
Reduction of *L. monocytogenes* attachment to uncoated (uc) and coated (c) stainless-steel discs after inoculation by applying *L. monocytogenes* as suspension (**a**), by pressing (**b**) and by sliding (**c**). The mean values (± SD) from four (**a** and **c**) and five (**b**) replicates are shown

Attachment after inoculation by liquid suspension was similar for both surfaces. On uncoated discs, an average of 1.020 log_10_ cfu was unable to attach to the surface while on coated discs, 1.155 log_10_ cfu did not attach (*p* = 0.288; Fig. 1a). The average of cells that were unable to attach by application as pressing was slightly higher on coated (2.514 log_10_ cfu) than on uncoated discs (2.320 log_10_ cfu), but not statistically significant (*p* = 0.626; Fig. 1b). Application as gliding seems more than 1 log higher on coated (3.568 log_10_ cfu) than on uncoated surfaces (2.430 log_10_ cfu) but not statistically significant due to high SD (*p* = 0.101; Fig. 1c).

### Growth

**Fig. 2:**
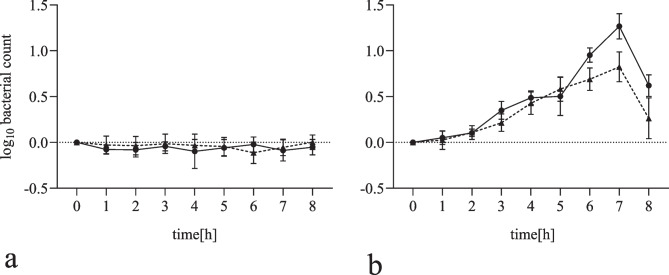
Growth of *L. monocytogenes* on uncoated (●) and coated (▲) stainless-steel discs at 10 °C and 85–90% humidity (**a**) and at 30 °C and 75–80% humidity (**b**). The mean values (± SD) from six replicates are shown

Growth of *L. monocytogenes* at 10 °C and 85–90% humidity was investigated. Neither a growth nor a loss rate could be detected on both surface types over 8 h compared to the initial bacterial count (Fig. 2a). Additionally, the growth capacity was also tested at 30 °C and 75–80% humidity. Growth of *L. monocytogenes* was observed on average on uncoated (approx. 1 log_10_ stage) and coated (approx. 0.5 log_10_ stage) until the seventh hour. No further growth was observed afterwards (Fig. 2b). No statistical significance could be found at either 10 or 30 °C due to the high standard deviations.

### Detachment

**Fig. 3:**
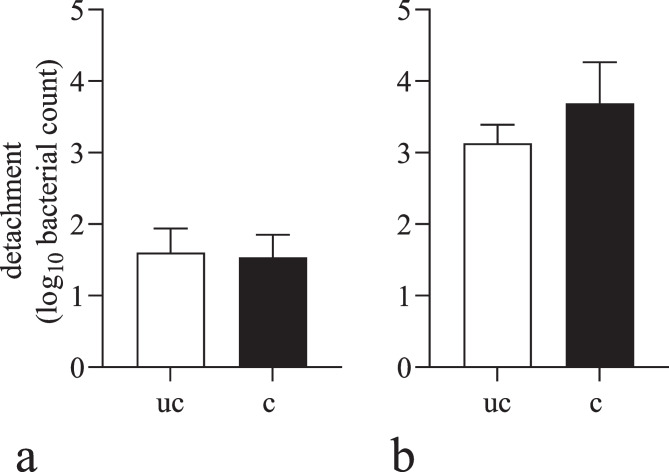
Detachment of *L. monocytogenes* on uncoated (uc) and coated (c) stainless-steel discs by distilled water (**a**) and by detergent foam (**b**). The mean values (± SD) from three replicates are shown

Cleaning with hot distilled water led to a similar rate of detachment from uncoated and coated stainless-steel surfaces (1.533 vs. 1.600 log_10_ cfu, respectively, *p* = 0.817; Fig. 3a), while cleaning with detergent foam led to a higher detachment of 3.130 log_10_ cfu from uncoated and 3.690 log_10_ cfu from coated stainless-steel surfaces. However, the difference was not statistically significant in regard of coating (*p* = 0.199; Fig. 3b).

## Discussion

This study examined the attachment ability, growth behaviour and detachment ability of *L. monocytogenes* on silica-coated compared to uncoated stainless-steel surfaces. The interactions between microorganisms and nanoscale surfaces are complex physicochemical processes (Feng et al. 2015). The preceding non-specific and reversible attachment is characterised by electrostatic forces, van der Waals forces and hydrophobic interactions. It is followed by the specific and irreversible attachment, which is marked by the beginning production of extracellular polymeric matter. Through this matrix, the adherent microbial cells form a complex with the surface and become protected from environmental influences (Bayoudh et al. 2006; Garrett et al. 2008; Palmer et al. 2007). While reversible adhesion removes bacteria by minimal force, irreversible adherent cells can only be removed by strong mechanical forces, chemical agents or heat (Chmielewski and Frank 2006; Sinde and Carballo 2000). The incomplete disruption of adhesion means that adherent cells can persist even after cleaning and disinfection and consequently pose a serious risk to the food industry through cross-contamination. Several studies observed that irreversible adhesion begins after 20 min and at the latest after 4 h at a temperature of 4–20 °C (Hood and Zottola 1995; Smoot and Pierson 1998).

Short incubation times of 5 to 20 min in the presented three attachment experiments lead to the assumption that attachment can be classified as reversible and the applied cells should detach (Mittelman 1998). The observed tendency to reduced attachment between uncoated and silica-coated stainless-steel surfaces could have resulted from the different surface topography. Zeraik and Nitschke (2010) determined that the cell adhesion decreases on a surface that becomes more hydrophobic through appropriate surface conditioning. Applying the silicon dioxide coating increases the hydrophobicity of the stainless-steel surface, which is confirmed by a contact angle measurement according to Schumann-Muck et al. (2023). The water contact angle determined for stainless-steel surfaces without a silicon dioxide coating was 56.7 ± 2.8° and for stainless-steel surfaces with a silicon dioxide coating 115.4 ± 1.5°. Vogler (1998) classified surfaces with a water contact angle < 65° as hydrophilic and with > 65° as hydrophobic. Accordingly, the applied silica coating leads to a reduced contact area between inoculum and surface. The same cell number competes for fewer binding spots on the surface compared to uncoated stainless-steel surfaces with a larger contact area (Palmer et al. 2007). Less cells can consequently attach and can be recultured.

Incubation times of the growth experiments ranged from 60 min to 8 h, which means irreversible attachment according to the criteria of Mittelman (1998). No growth could be detected at an incubation time of 8 h under the given conditions of 10 °C and 85% humidity. These less-than-optimal environmental settings for *L. monocytogenes* are representative of the growth conditions in food-producing and cutting plants. The measurement period of 8 h is based on a working shift in food-production plants with subsequent cleaning. It seems that the cells need significantly longer for replication which is consistent with previous studies. Other research groups have previously observed that generation times of *L. monocytogenes* could be between 5 and 14 h at 8 °C or longer than 24 h at 10 °C (Brierley et al. 2020; Cordero et al. 2016; Marshall and Schmidt 1988). Coating seems to have no influence on the growth of *L. monocytogenes* at 10 °C, because although there were fewer binding spots due to a lower water contact angle, a similar number of cells could be regenerated compared to the uncoated surfaces.

Growth of *L. monocytogenes* over a period of 8 h with an optimal growth temperature of 30 °C was very similar for uncoated and coated stainless-steel surfaces (Gray and Killinger 1966). Generally, a growth was expected since the generation time of *L. monocytogenes* is only 45 min at 30 °C (Petran and Zottola 2006). The results also support the hypothesis of competition for the available binding spots on the stainless-steel surface, as slightly fewer cells were always recultivated from the coated stainless-steel surfaces. The continuous increase in cell numbers up to hour 7 also suggests that nutrients were available to the applied cells. The added bovine serum albumin, which was intended to simulate protein contamination of the stainless-steel surfaces, or the inoculum could have served as a nutrient base. A decrease of cell number between hour 7 and 8 could indicate a deficiency of nutrients and beginning of cell degeneration.

The inoculation time of the experiments to test the detachment behaviour was 5 min. According to this, as with attachment, it was a reversible cell adhesion (Mittelman 1998). A treatment with distilled water detached nearly the same number of *L. monocytogenes* from both surfaces, while the detergent tended to detach more bacteria from coated stainless-steel discs. The results with detergent confirm the general assumption that less cells can bind to the stainless-steel surface due to the silica coating and the smaller contact area between inoculum and surface. This water-repellent effect on nanostructured surfaces with silica has already been described by Singh et al. (2011). It leads to a self-cleaning effect of the surface. However, it is also possible that the limited number of binding spots is occupied by proteins. Palmer et al. (2007) have described that proteins compete with microbial cells for binding sites on a surface. Protein additions can also reduce bacterial adhesion to nanostructured surfaces and create a passivation effect. A flattening effect was also observed due to the protein layer formed, which could reduce the function of the nanoscale coating (Singh et al. 2011). The observed flattening effect could confirm our findings, as the added bovine serum albumin could inhibit the hydrophobic effect of the nanoscale silica coating, making it more difficult for the microbial cells to detach. The results of the present study are also consistent with previously published results of detachment of different microorganisms from silica-coated stainless-steel surfaces (Schumann‐Muck et al. 2023).

The present study neglected chemical-physical surface parameters such as surface topography, charge and energy as well as specific coating properties such as roughness, thickness and structure, which also influence bacterial adhesion. The low impact of the tested silica coating could result from the low incubation times of the laboratory experiments (5 to 20 min and 60 min to 8 h), which reflect the practical conditions in food-production plants. Furthermore, the study was a quantitative determination of the total recultivable *L. monocytogenes*. Therefore, especially the VBNC (viable but nonculturable) status described for *L. monocytogenes* should be considered in future studies using a detection method such as PMA-q-PCR (Pan and Breidt 2007; Wideman et al. 2021).

## Conclusion

Modifying stainless steel with a nanoscale silica coating tends to lead to less attachment and better detachment and does not seem to influence bacterial growth of *L. monocytogenes*. The results suggest that surface modification of stainless steel with silica is not sufficient to prevent cross-contamination. A persistence of *L. monocytogenes* in food-producing plants is even probable with a surface modification since growth is not influenced. The selected silicon-dioxide coating for stainless-steel surfaces cannot be recommended for targeted use in bacterial reduction for *L. monocytogenes* at the present time.

It is possible that the coating will be more effective on other surface materials due to modifications in surface parameters (such as roughness, thickness or structure). Future studies could therefore investigate the coating on other surfaces from food-producing plants, for example rubber or plastic.

## Data Availability

The datasets generated during and/or analysed during the current study are available from the corresponding author on reasonable request.
